# Assessment of the root and canal morphology in the permanent dentition of Saudi Arabian population using cone beam computed and micro-computed tomography – a systematic review

**DOI:** 10.1186/s12903-024-04101-3

**Published:** 2024-03-16

**Authors:** Mohammed Mustafa, Rumesa Batul, Mohmed Isaqali Karobari, Hadi Mohammed Alamri, Abdulaziz Abdulwahed, Ahmed A. Almokhatieb, Qamar Hashem, Abdullah Alsakaker, Mohammad Khursheed Alam, Hany Mohamed Aly Ahmed

**Affiliations:** 1 Department of Conservative Dental Sciences, College of Dentistry, Prince Sattam Bin Abdulaziz University, Al-Kharj, 11942 Saudi Arabia; 2 Conservative Dentistry Unit, School of Dental Sciences, Universiti Sains Malaysia, Health Campus, Kubang Kerian 16150, Kelantan, Malaysia; 3 Dental Research Unit, Center for Global Health Research, Saveetha Medical College and Hospital, Saveetha Institute of Medical and Technical Sciences, Chennai, 600077 Tamil Nadu India; 4 Department of Restorative Dentistry & Endodontics, Faculty of Dentistry, University of Puthisastra, Phnom Penh, 12211 Cambodia; 5 Consultant Endodontics, Department of Dentistry, King Faisal Specialist Hospital and Research Center, Riyadh, Saudi Arabia; 6 Consultant endodontist, Department of endodontics, Prince Abdulrahman Advanced Dental Institute, Ministry of defence, Riyadh, Saudi Arabia; 7 Department of Preventive Dentistry, College of Dentistry, Jouf University, Sakaka, 72345 Saudi Arabia; 8 Department of Restorative Dentistry, Faculty of Dentistry, Universiti Malaya, Kuala Lumpur, Malaysia

**Keywords:** CBCT, Dental anatomy, Dental diagnostic imaging, Dental pulp, Endodontics, Micro-CT, Morphology, Root, Root canal

## Abstract

**Introduction:**

Root canal treatment procedures require a thorough understanding of root and canal anatomy. The purpose of this systematic review was to examine the morphological differences of teeth root and their canals assessed using cone-beam computed and micro-computed tomography in Saudi Arabian population.

**Methodology:**

An electronic search was conducted in PubMed / Medline, Scopus, Google Scholar, and Web of Science databases until January 2023 to retrieve related studies. “Root canal morphology,” “Saudi Arabia,” “Micro-CT,” and “cone-beam computed tomography” were used as keywords. A modified version of previously published risk of bias assessment tool was used to determine the quality assessment of included studies.

**Results:**

The literature search revealed 47 studies that matched the criteria for inclusion, out of which 44 studies used cone beam computed tomography (CBCT) and three were micro-computed tomography (micro-CT) studies. According to the modified version of risk of bias assessment tool, the studies were categorized as low, moderate, and high risk of bias. A total of 47,612 samples were included which comprised of either maxillary teeth (5,412), or mandibular teeth (20,572), and mixed teeth (21,327). 265 samples were used in micro-CT studies while 47,347 teeth samples were used in CBCT studies. Among the CBCT studies, except for three, all the studies were retrospective studies. Frequently used imaging machine and software were 3D Accuitomo 170 and Morita’s i-Dixel 3D imaging software respectively. Minimum and maximum voxel sizes were 75 and 300 μm, Vertucci’s classification was mostly used to classify the root canal morphology of the teeth. The included micro-CT studies were in-vitro studies where SkyScan 1172 X-ray scanner was the imaging machine with pixel size ranging between 13.4 and 27.4 μm. Vertucci, Ahmed et al. and Pomeranz et al. classifications were applied to classify the root canal morphology.

**Conclusion:**

This systematic review revealed wide variations in root and canal morphology of Saudi population using high resolution imaging techniques. Clinicians should be aware of the common and unusual root and canal anatomy before commencing root canal treatment. Future micro-CT studies are needed to provide additional qualitative and quantitative data presentations.

## Introduction

The aim of an endodontic therapy is adequate cleaning and shaping of the root canals to eliminate microorganisms, filling of the canal three-dimensionally with an inert material to create a hermetic seal and placing a coronal restoration to avoid communication between peri-radicular tissues and the oral environment [1, 2]. However, the anatomy of the root canal is complex, that may vary from straight to curved canals or simple to more complex configurations in addition to wide range of variations in accessory canals and apical foramen anatomy [3, 4]. Such anatomical variations may vary among gender, sex and ethnicity [5]. Untreated or missed canals has an impact on the outcome of an endodontic procedure [1, 6]. Therefore, adequate knowledge and meticulous understanding of the normal and unusual anatomy of human teeth are essential to avoid possible complications and failure of root canal therapy [7].

Over the decades, several invasive and non-invasive laboratory methods have been introduced to study the root and canal anatomy. An ideal method to study the root anatomy should be simple, accurate, non-destructive, and reproducible [8, 9]. Staining and clearing technique [10], scanning electron microscopy [11], 2D radiographic imaging [12] and 3D tomographic scanning including cone beam computed tomography (CBCT) [13] and micro-computed tomography (micro-CT ) [3] are the main laboratory methods to study root canal anatomy. In addition to this *in-vivo* methods include clinical studies consisting the clinical detection of root canals during the procedures [14] and observational CBCT clinically on various groups [15].

After reporting the pivotal role of radiography for determining the root canal length in 1899 by Kell, maxillofacial CBCT a 3D imaging technique was introduced in 1996 and was approved for dental use in 2001 by Food and Drug Administration (FDA) in the United States [16, 17]. It is a method obtained from computed tomography (CT) which provide data that is composed of identical dimensions at all sides permitting precise measurement of objects, hence, provide superior 3D images from received data with comparatively lesser radiation dose and time [18, 19]. The field of view is less but they are less invasive compared to conventional CT devices. Greater advantage of this technique is it construct the images in all three planes i.e., sagittal, axial and coronal, additionally images can be revolved in any plane avoiding superimposition and data can be reconstructed in their original spatial associations [17, 20].

Similarly, micro-CT has gained popularity in endodontics due to its non-invasive, reproducible and non-destructive nature. It accurately gives the details of root canal morphology with higher resolution [21, 22]. Micro-CT demonstrates a wide range of qualitative and quantitative analysis of the internal and external tooth anatomy [23].

Various studies have been conducted on different populations to understand the root canal anatomy [7, 24–27]. Similarly, several studies examined the root and canal anatomy of Saudi population revealed the morphological variations in permanent maxillary and mandibular incisors [28–30], maxillary first and second premolars [31], mandibular premolars [32, 33], maxillary and mandibular first and second molars [34]. Numerous studies are conducted on Saudi Arabian and sub–Saudi Arabian population. Hence, a systematic review would help to combine different studies and provide beneficial data that can guide clinically and in the research for better outcome.

This systemic review aimed to evaluate the root canal morphology of human permanent teeth in Saudi population using CBCT and micro-CT technology.

## Methodology

### Protocol

The review adhered to PRISMA guidelines proposed for reporting of systematic reviews and quantitative analysis (http://www.prisma-statement.org, accessed on 25 February 2023). This current systematic review is registered in the Open Science Framework database (https://osf.io/) dated February 1, 2022, with the registration number (10.17605/OSF.IO/FX8RN).

### Research question

The search strategy was carried out using the condition, context, and population framework [35, 36]. The following question is considered: “What is the prevalence and variation of root canal configurations in the permanent dentition of the Saudi Arabian population, as assessed by CBCT and micro-CT studies?” Studies that only used CBCT and micro-CT were included. The context comprised of the studies conducted using CBCT and micro-CT on Saudi Arabia population.

### Search strategy

A comprehensive electronic search was carried out in electronic databases, PubMed / Medline, Scopus, Cochrane, Embase and ScienceDirect without any restriction on the year of study until January 2023. Relevant articles were identified through manual searching of reference lists from included studies and relevant review articles. Additionally, we searched the tables of contents of key journals in endodontics and radiology to identify any potentially relevant articles that may not have been captured in the electronic searches. Gray literature includes unpublished or non-peer-reviewed sources such as conference proceedings, theses, dissertations, and reports. We conducted searches in databases such as OpenGrey, ProQuest Dissertations & Theses Global, and Google Scholar to identify any relevant gray literature related to our research question. Additionally, we searched relevant conference proceedings and contacted experts in the field to identify any additional gray literature sources. The number of articles retrieved from each database is presented in Table 1. Medical Subject Heading (MeSH) terms, keywords and other free terms combined with Boolean operators (OR, AND) were used for searching articles on PubMed, Scopus, Cochrane, Embase and ScienceDirect databases. Endnote X8 software imported the literature search results and removed duplicates. Abstracts were screened to check for eligibility criteria. Full articles of those that cleared were retrieved.

**Table 1:** Search strategies using keywords

Database	Search Strategies	Results
PubMed	((((((Root canal anatomy) OR (Root canal morphology)) OR (Root canal configuration)) ) AND (Cone-beam computed tomography)) OR (Micro-CT)) AND (Saudi Arabia)	271
Scopus	Root canal anatomy OR Root canal morphology OR Root canal configuration AND Cone-beam computed tomography OR Micro-CT AND Saudi Arabia	158
Cochrane	“Root canal anatomy” OR “Root canal morphology” OR “Root canal configuration” AND “Cone-beam computed tomography” OR “Micro-CT” AND “Saudi Arabia”	21
ScienceDirect	(Root canal anatomy OR Root canal morphology OR Root canal configuration) AND (Cone-beam computed tomography OR Micro-CT) AND (Saudi Arabia)	08
EMBASE	“Root canal anatomy” OR “Root canal morphology” OR “Root canal configuration” AND “Cone-beam computed tomography” OR “Micro-CT” AND “Saudi Arabia”	08
Total		466

### Data extraction

Two researchers (M.I.K and M.M) performed an electronic literature search and assessment for inclusion was done on 05 February 2023, using MeSH terms and keywords, as well as the Boolean operators “OR” and “AND” to compile relevant material using appropriate filters. Followed by data extraction and quality assessment. In case of disagreement, the third author (HMA) was consented and finalized. The keywords used were. “Root canal anatomy”, “Root canal morphology”, “Root canal configuration”, “Cone-beam computed tomography”, “Micro-CT” and “Saudi Arabia”. The required literature was then gathered using proper filters by combining these key terms with the Boolean operators “OR” and “AND” as shown in Table 1.

### Eligibility criteria

**Fig. 1:**
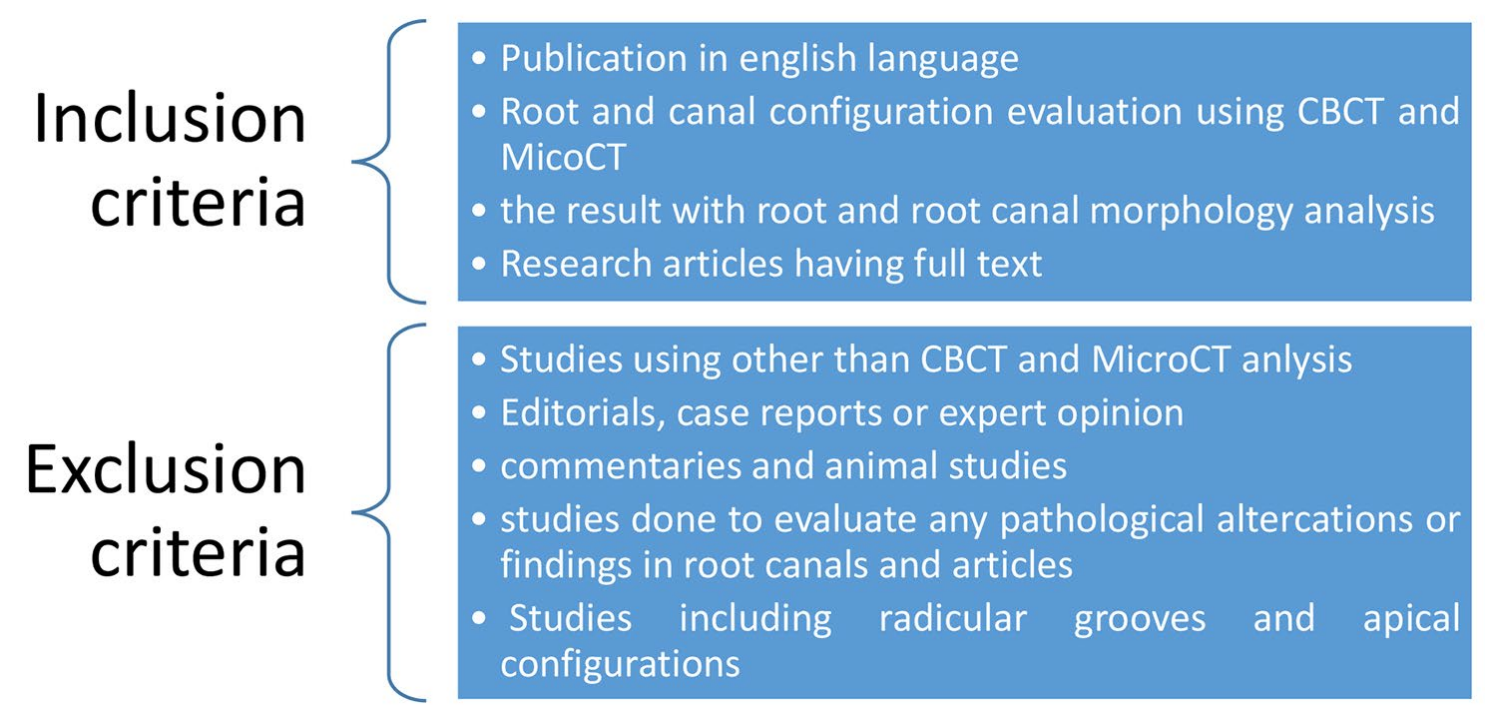
Criteria for Inclusion and exclusion of studies

Cross-sectional studies assessing root and canal morphology of permanent dentition using CBCT and micro-CT amongst Saudi Arabia population were included in this study. Editorials, case reports or expert opinion, commentaries, animal studies, studies done to evaluate any pathological altercations or findings in root canals and articles written in a language other than English were excluded Fig. 1. We did not seek additional information or clarification from authors of included studies.

### Risk of bias assessment

Two reviewers (M.I.K and R.B) evaluated the studies based on the modified version of earlier risk of bias assessment tool [37]. The included assessment is categorized as yes (adequate), unclear (not specified) and no (inadequate). The assessment was consisting of the following objectives:

i. Sample size calculation (yes, no, and unclear): calculation of sample size is essential for any research as adequate sample size is important for generalizing the results and obtaining a valid conclusion. Sample size calculator and G* POWER can be used to calculate the sample size.
ii. Reporting and quality of data: various factors affect the outcome of an image like voxel size, x-ray machine and software used. Hence when all the parameters are present it implies that the study is adequate.
iii. Result description (yes, no, and unclear): main features in the results included the assessment of root canal configuration using different root canal classifications, detection of number of roots, canals, and accessory canals. Since most of the included studies in the present systematic review are CBCT studies, the outcome generally contained the above-mentioned features.
iv. Reliability of an observer (yes, no, and unclear): calibration of data is essential to minimize human error and to validate the results by reducing the potential bias. The trained observers are involved in this process. Intra-observer reliability is when a single observer conducts and repeats the same assessment whereas, inter-observer reliability is when two observers are involved in the same assessment at a specific time. Cohen’s kappa test is considered as the valuable tool for this task.

Attrition bias (yes, no, and unclear): reporting of sample loss, proper sampling technique and sampling methods used in the study so that to cover the mentioned population. It highlights whether the population is regional or central. Attrition bias is not about the tooth loss, but it features about the population that belongs to specific region rather than generalizing the population.

**Fig. 2:**
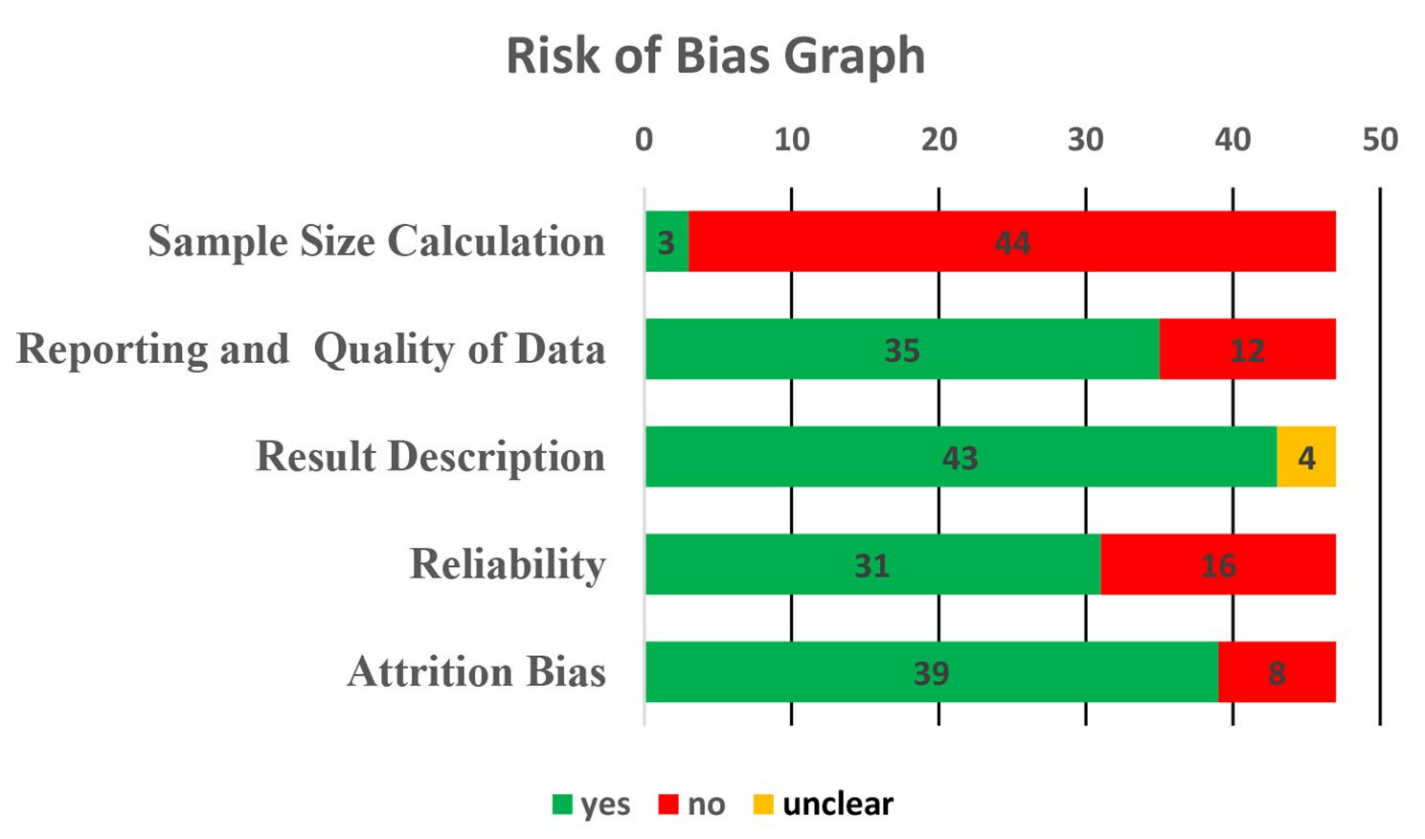
Risk of bias analysis of the 47 studies included in this review

A total of 47 articles were eligible to be the part of this analysis. Data were independently extracted by two authors (M.I.K and R.B) and are summarized in Fig. 2.

**Fig. 3:**
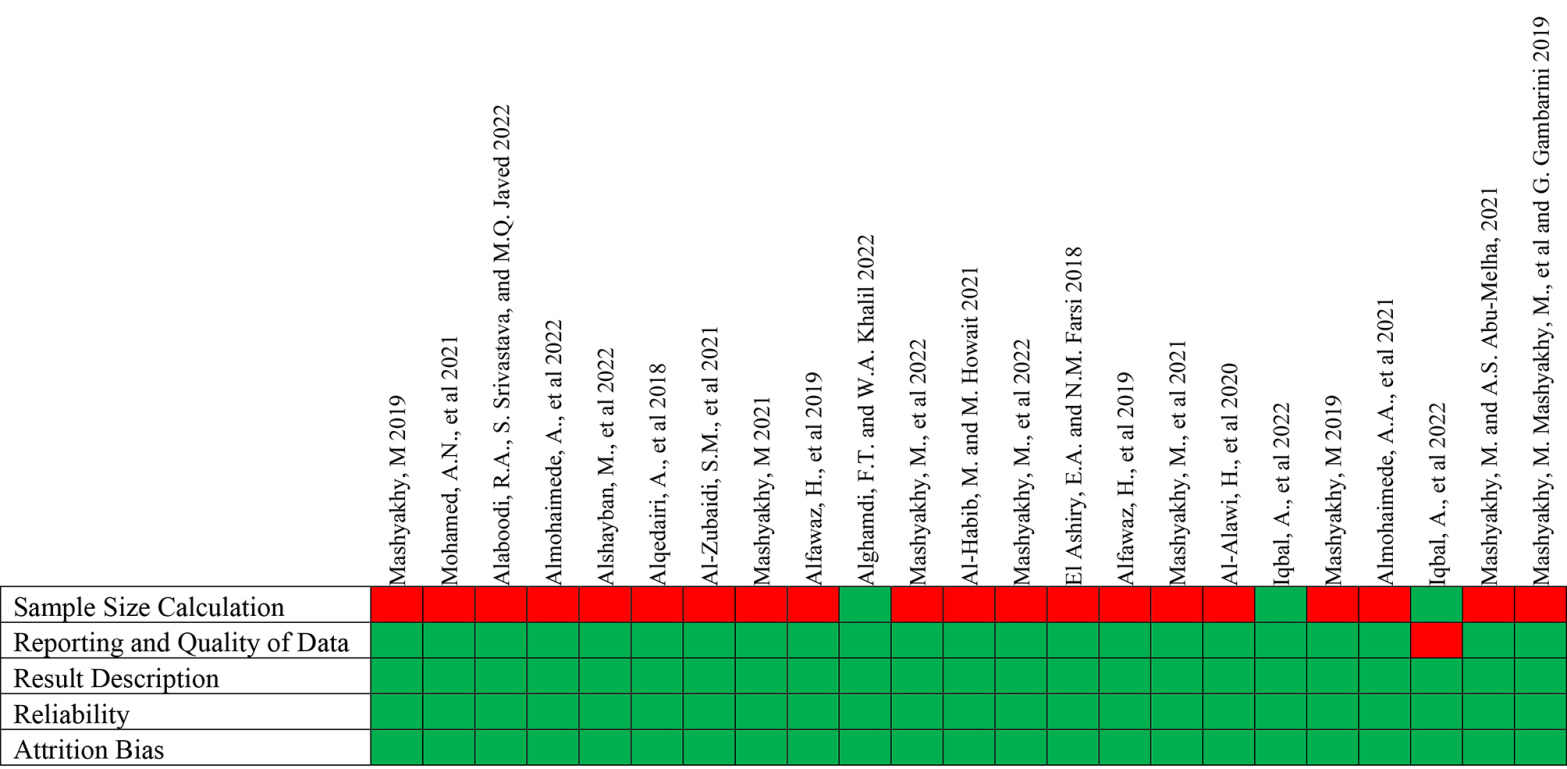
Low risk of bias: studies that met at least four of the criteria (Green-Yes; Red-No)

**Fig. 4:**
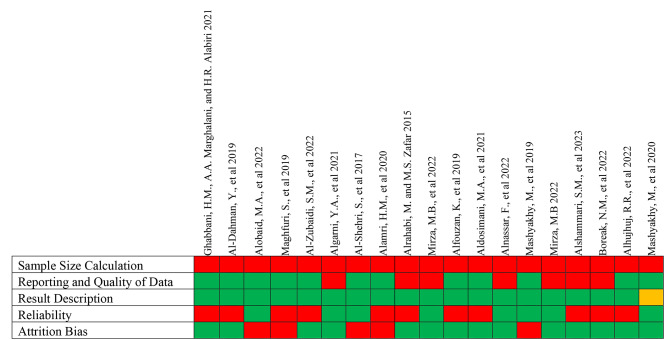
Moderate risk of bias: studies that met between two and four of the criteria (Green-Yes; Red-No; Yellow-Unclear)

**Fig. 5:**
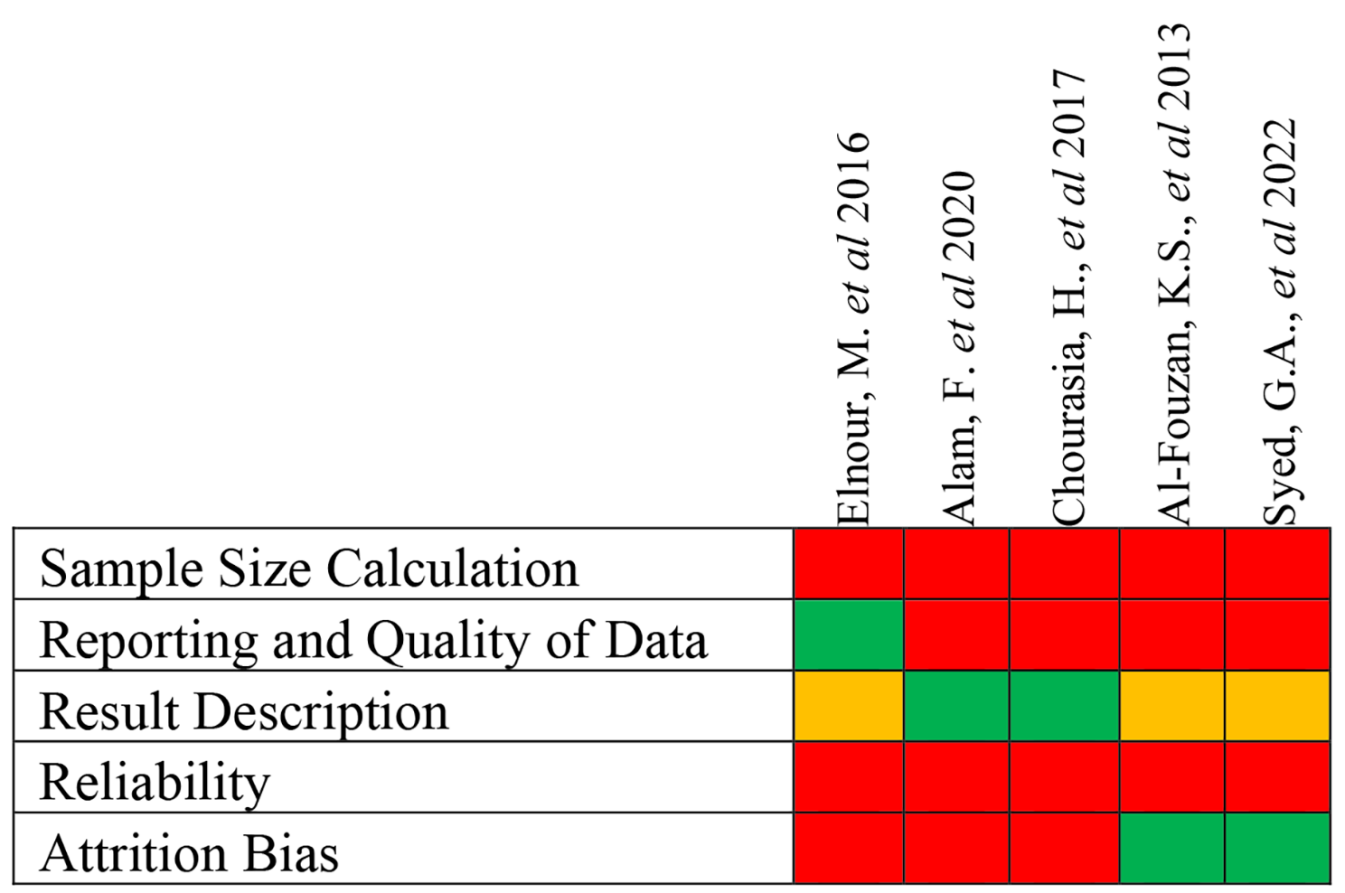
High risk of bias: studies that met less than two criteria (Green-Yes; Red-No; Yellow-Unclear)

Further to justify the studies they were classified as follows:

A. Low risk of bias (i.e., studies meeting at least four of the assessment criteria): Fig. 3 represents the studies that met at least four of the quality criteria.
B. Moderate risk of bias (i.e., studies meeting the criteria between two and four): Fig. 4 displays the studies that met the assessment criteria between two and four.
C. High risk of bias (i.e., studies meeting less than two assessment criteria): Fig. 5 shows the data of studies that met less than two quality criteria and are classified as high risk of bias.

## Results

### Outcome of study selection

**Fig. 6:**
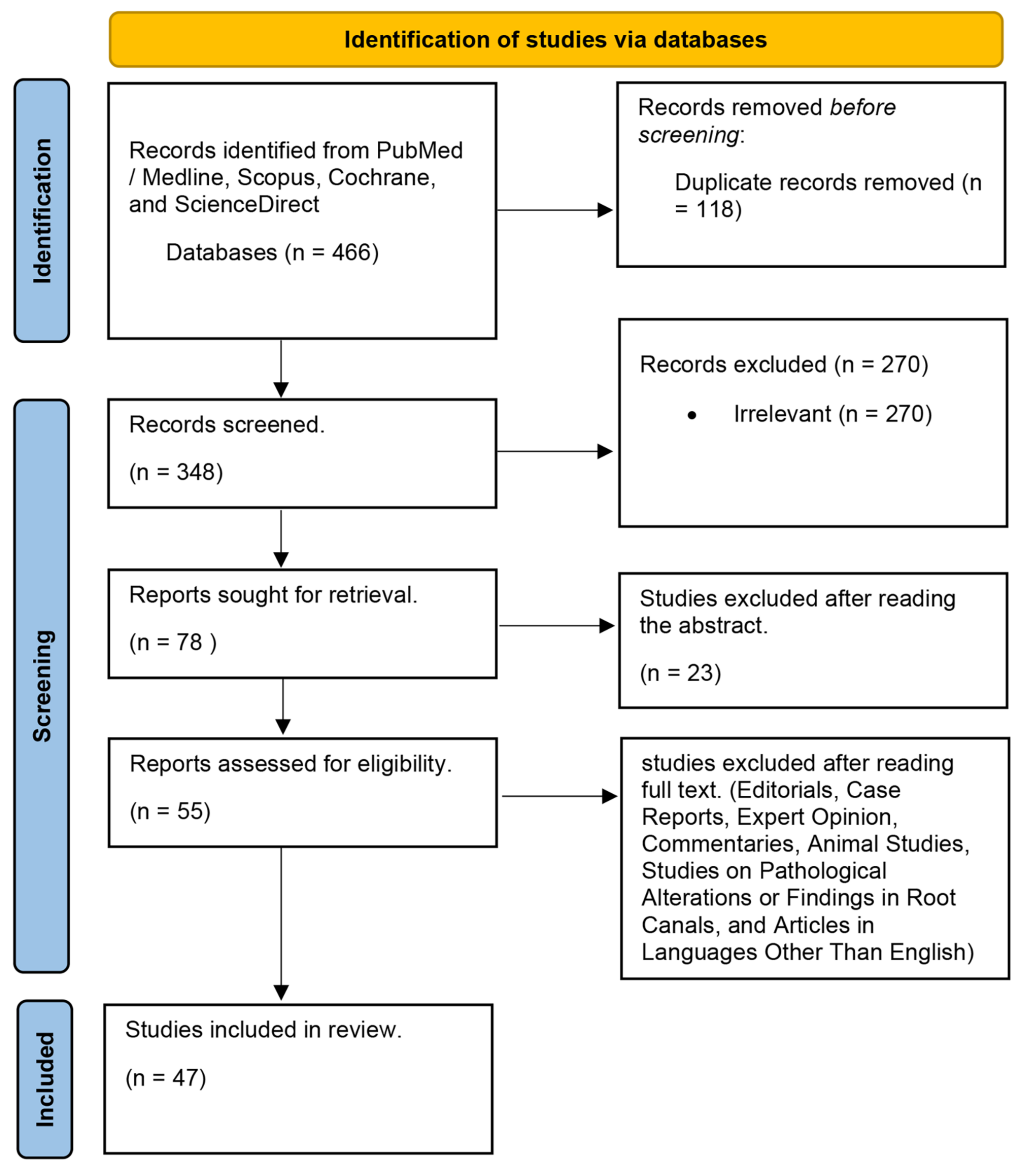
PRISMA flowchart

Cross-sectional studies evaluating root and canal morphology of permanent dentition in Saudi Arabia population using CBCT and micro-CT were included in this study. Editorials, case reports or expert opinion, commentaries, animal studies, studies done to evaluate any pathological altercations or findings in root canals and articles written in a language other than English were excluded. Studies that used the different diagnostic tools other than CBCT and micro-CT were excluded. Further four rounds of screening were performed, where the first selection process deleted the duplicate articles, next unrelated articles were screened out through titles. In third round the abstracts were read to screened out the unrelated articles and finally the unrelated articles were screened out after reading the full text. Figure 6 shows the selection criteria as it follows the PRISMA guidelines.

### Study charateristics

**Fig. 7:**
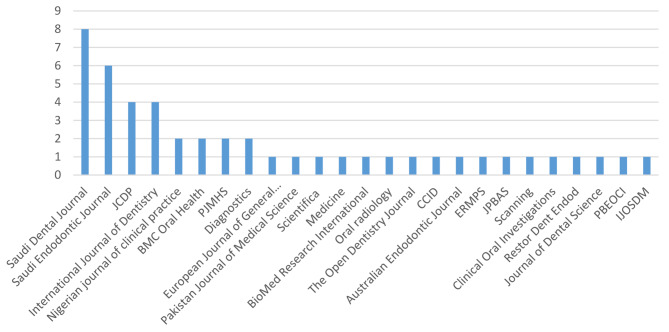
Included articles published in the respective journals Where, JCDP: The Journal of Contemporary Dental Practice; PJMHS: Pakistan Journal of Medical & Health Science; CCID: Clinical, Cosmetic and Investigation Dentistry; ERMPS: European Review for Medical and Pharmacological Sciences; JPBAS: Journal of Pharmacy and Bio allied Sciences; PBEOCI: Pesquisa Brasileira Em Odontopediatria Clinica Integrada and IJOSDM: International journal of oral science and dental medicine

All the included 47 studies were carried out in the different regions of Saudi Arabia lasted for different time periods between 2013 and 2022 and were issued in various journals as listed in Fig.  7.

#### Number of teeth

Total number of samples involved and studied was 47,612, out of which 265 samples were used in micro-CT studies and 47,347 teeth were used in CBCT studies. Among the included micro-CT studies, maxillary second premolars were 100, 100 were maxillary first molars and 65 were maxillary first and second molars. The studies incorporated either maxillary teeth (*n* = 5,412) or mandibular teeth (*n* = 20,572) and studies including both upper and lower teeth were (*n* = 21,327). Among the total maxillary teeth, 657 were maxillary first molars, 351 were maxillary second molars. In case of mandibular teeth 8,603 were mandibular incisors, 316 were mandibular first premolar, 2400 were mandibular second premolar and 915 were mandibular first molars. Excluding the above results, few studies had mixed samples such as maxillary first and second premolars were 2362, maxillary first and second molars were 1,580, mandibular first and second premolars were 4014, mandibular first and second molars were 3,389 and 1433 were mandibular premolars and molars.

#### In-vivo studies

A total of fourty one in-vivo studies (CBCT studies) were included in the current systamatic review.

#### In-vitro studies

Six studies were in vitro where three studies used CBCT and three studies were performed using micro-CT. Storage media in CBCT studies was 2.5% sodium hypochlorite, 10% formalin and normal saline. While, 10% formalin and normal saline were used in micro-CT studies.

#### Scanning machines and software

##### CBCT- *in-vivo* studies

Different CBCT scanning machines were used to analyze the teeth among which maximum studies used 3D Accuitomo 170 (9) and CS9300 3D imaging machine in nine studies followed by Planmeca promax 3D Max digital imaging Device in seven studies, iCat imaging machine in four studies, Scanora 3D equipment in four studies, Galileos ComfortPlus Asystem Sidexex in two studies, CS 8100 3D machine in two studies and KAVO OP 3D Pron in one study. Various software were employed for interpretation of data. Morita’s i-Dixel 3D imaging software was applied in eleven stdies,, Planmeca Romexis viewer software in nine studies, On Demand 3D software in five studies, CS 3D imaging software in four studies, Blue Sky Plan in two studies, Sidexis XG software was included in two studies and vision software in one study.The minimum and maximum voxel size was 75 to 300 μm. In order to classify the root and canal morphology of involved teeth in the studies, Vertucci’s classification [38] was frequently applied (31) followed by the new coding system introduced by Ahmed et al. [39] in six studies, Pomeranz et al. classification [40] in two studies, Fan et al. [41] in one study, Calesen and Alexandersen [42] and Song et al. classification [43] in one study.

##### CBCT- *in-vitro* studies

3D Accuitomo 170 scanning machine was used in one CBCT laboratory study while no data of machines was mentioned in other two studies. i-Dixel 3D imaging and CS 3D imaging software were applied in two in vitro studies although one study did not specify the software used. The voxel size ranged from 76 to 125 μm All the three *in_vitro* CBCT studies used Vertucci classification.

##### Micro-CT studies

SkyScan 1172 micr-CT scanner was used in all the three studies. SkyScan CT-Volume v2.2 (Bruker Corp., Antwerp, Belgium) software, (SkyScan 1172, SkyScan, Bruker, Belgium and CTAn software (Bruker microCT) software were used in the three respective studies. The voxel size was in the range of 13.4 to 27.4 μm. Out of the three studies, one study used Vertucci classification and other study samples were classified using Pomeranz et al. classification (Tables 2, 3 and 4).

**Table 2:** Characteristics of the included CBCT in-vivo studies

Study Reference	Sample Size	Sample type	CBCT machine	Voxel size (Resolution)	Software used	Classification System	Technique	Results	Conclusion
[47] Ghabbani, H.M., A.A. Marghalani, and H.R. Alabiri 2021	1624	Mandibular incisors	CS 9300 PREMIUM 3D (Carestream, Rochester, NY, USA)	0.3 mm	Blue Sky Plan	Vertucci’s classification	Retrospective data from database	Study found that 24.6% of mandibular central incisor had type I configuration then type III in 21.5% followed by type V in 2.4% of teeth, while the mandibular lateral incisor had 25.6% type I, 20.6% type III and 7.1% type V. Occurrence of type IV and VII was less, on the other hand type II, VI and type VIII were absent. With regards to gender females had 64.8% type I canals followed by 29.4% type III root canals, whereas 46.9% type III configuration was showed by males’ teeth then 45.2% of type I canal configuration.	Study concluded that all the mandibular incisors in the study had one root and increased incidence of one canal configuration. Canal configuration revealed bilateral symmetry in majority of mandibular incisors.
[48] Mashyakhy, M 2019	822	Mandibular incisors	3D Accuitomo 170 (MORITA, Japan)	0.25 mm	Morita’s i‑Dixel 3D imaging software	Vertucci’s classification	Retrospective data from database.	Findings in the central incisors are as follows: One root in 100% teeth, one canal in 73.7%, two canals in 26.3%, type Iin 73.7% and type III in 26.3%. Lateral incisor had one root in 99.5% teeth, two roots in 0.5%, one canal in 69.2%, two canals in 30.8%, type I in 69.2%, type III in 29.8% and type V configuration in 1.0% of teeth. Both the incisors had no statistical difference, but gender difference was seen in central incisor while it was absent in lateral incisor.	Few canals and their configuration showed bilateral asymmetry which can be of clinical importance.
[49] Mohamed, A.N., et al. 2021	376	Mandibular incisors	Galileos ComfortPLUS System Sidexis	0.5 mm	SIDEXIS XG (Sirona 3D, Germany)	Vertucci’s classification	Retrospective data from database.	It was discovered that all the lower incisors had one root and occurrence of canal configuration is: type I-44.4%, type II-8%, type III-44.9% and type V was 2.1%.	Study concluded that majority of mandibular canines had one root and type IIIcanal configuration.
[50] Alaboodi, R.A., S. Srivastava, and M.Q. Javed 2022	928	Mandibular incisors	GALILEOS Comfort CBCT machine (Dentsply- Sirona Dental Systems, Montagematerial, Galileos, SK, Bensheim, Germany)	0.3-0.15 mm	Sidexis XG 3D Viewer; Germany	Vertucci’s classification	Retrospective data from database	Study stated that all the lower incisors had one root and chances of more than one canal was 29.3% in CI and 38.57% in LI. Canal configuration in central incisor is: Type-I (70.6%); Type-III- (24.5%); Type-II (3.4%); Type-IV (0.9%) and Type-V- (0.5%). Type-VI, VII and VIII were absent. Lateral incisor had (61.6%) Type-I; Type-III (31.8%); Type-II (4.3%); Type-IV (0.8%); Type-V (1.3%) and Type-VII (0.2%). Type-VI and VIII were absent. Oval canals were present in 46.6% teeth and was higher in type III (80.1%) than type I (322%).	Although mandibular incisors had single root, but canals varied in terms of numbers and configuration along with presence of oval canal higher in type III configuration.
[51] Al-Dahman, Y., et al. 2019	454	Mandibular canines	CS9300 3D digital imaging system (Carestream, Rochester, NY) and Planmeca ProMax 3D (PLANMECA,	90–300 μm, ≤ 200 μm	Planmeca Romexis Viewer software (PLANMECA, Roselle, IL, USA)	Vertucci’s classification	Retrospective data from database	Study found that 99.8% of mandibular canines had one root and double root was seen in 0.2% which were men. 98% of females had one canal while 92.4% of single canal was seen in male. Predominant canal configuration was type I (95.4%) followed by type II (2.6%), type III (1.8%) and type IV (0.2%).	It was concluded that single root and canal with type I configuration was predominant finding although variations exist.
[52] Almohaimede, A., et al. 2022	1370	Permanent mandibular incisors	Planmeca ProMax 3D (PLANMECA, Roselle, IL, USA) and a CS9300 3D digital imaging system (Carestream, Rochester, NY)	75–600 μm	Planmeca Romexis Viewer software (Planmeca, Roselle IL)	Vertucci’s classification	Retrospective data from database	Central incisor had 100% one root and 99.9% of lateral incisor had one with 0.1% two roots. One canal was noted in 58.8% and two canals in 41.2% with no difference between central and lateral incisor but women had increased frequency of two canals (57.09%) than men (42.9%). Canal configuration is type I (58.83%), type III (28.24%), type II (6.4%), type V (5.76%) and type IV (0.72%).	It was evident from the results that Saudi population commonly had more than one canal and bilateral symmetry was more in central incisor than lateral incisor with respect to number of roots, canals and their configuration type.
[53] Alobaid, M.A., et al. 2022	1260	Mandibular central incisors	KAVO OP 3D Pron (KaVo Dental, Charlotte, NC, USA)	0.85 mm	On demand 3DTM imaging software (Cybermed Inc., Unit K Tustin, CA)	New system of classification	Retrospective data from database	All the right and left lower central incisors were single rooted on both the sides. Canal configuration in left central incisor: Male: 80.2% were (1-1-1), 13.6% had (1-2-1), 2.2% had (1-1-2), 3.7% had (1-2-2), and 0.4% were (2-2-1). Female: 84.4% were (1-1-1), 12.5% were (1-2-1), 2.5% had (1-2-2), and 0.6% were (2-2-1). Canal configuration in right central incisor: Male: 77.0% were (1-1-1), (1.1%) were (1-1-2), 16.3% were (1-2-1), 4.8% were (1-2-2), 0.4% were (2-1-1), and 0.4% were (2-2-1). Female: 84.9% had (1-1-1) configuration, 12.6% had (1-1-2), 2.0% were (1-2-2), and 0.6% had (2-2-1).	As per the study more observed classification was 1ManA1(82.6%) (Vertucci type I), then 1ManA1-2-1 was 13% (Vertucci type III). Canal configuration was symmetrical among the incisors with prevalence of single canal, but complex root morphology was also noted.
[54] Alshayban, M., et al. 2022	1769	Mandibular anterior	Planmeca Promax 3D Max Digital Imaging Device (Planmeca, Helsinki, Finland)	160 μm	Planmeca Romexis_ 3.6 viewer software (Planmeca, Helsinki, Finland).	Vertucci’s classification	Retrospective data from database	One root was 100% in lower central and lateral incisor while 98.4% of one root was seen in canine. Type I configuration was major among the lower anterior whereas 36.5% of type III was noted in central and 31% in lateral incisor. Regarding the sex, females had higher frequency of type I than type III while males had higher chances of type III.	Study concluded that majority of included Saudi population had single root and one canal while few central and lateral incisors were found with two canals.
[55] Alqedairi, A., et al. 2018	652	Maxillary first and second premolars	CS9300 3D digital imaging system (Carestream, Rochester, NY) and Planmeca ProMax 3D (Planmeca, Roselle IL).	90–300 μm and ≤ 200 μm	Planmeca Romexis Viewer software (Planmeca, Roselle IL).	Vertucci’s classification	Retrospective data from database.	Predominantly maxillary first premolar was double rooted (75.1%) with type IVcanal configuration (69.1%) whereas second premolar was single rooted (85.2%) with type I (49.4%) canal system. Excluding type VII configuration in second premolar, all types were seen in both the premolars.	Researchers concluded that one root and type IV canal configuration is prevalent in first maxillary premolar however two roots and type 1 was common in second premolar. First premolars had one canal (21.3%), two canals (75.4%), three canals (3.3%) apically whereas second premolar exhibited one canal (80.2%), two canals (18.9%), and three canals (0.9%) apically.
[56] Al‑Zubaidi, S.M., et al. 2021	1000	Maxillary first and second premolars	Carestream CS 8100 3D (Carestream Dent LLC, Atlanta, USA).	75 μm	CS 3D Imaging Software (Carestream Dent LLC, Atlanta, USA).	Vertucci’s classification	Retrospective data from database.	It was discovered that out of 500 first maxillary premolars, 39.8% had single roots, 58.6% were double rooted followed by three roots (1.6%). Most common root canal configuration was type IV (57.8%) then type II (32.8%). 83.2% of second maxillary premolar had single root followed by two roots (15.8%) and three roots (1.0%). Type Iconfiguration was common in second premolar (60.4%) then type II (16.4%).	Two canals and two roots were major finding in first maxillary premolars while one canal and single root was common in maxillary second premolar.
[57] Mashyakhy, M 2021	710	Maxillary first and second premolars	3D Accuitomo 170 (MORITA, Japan)	0.25 mm	Morita’s i‑Dixel 3D imaging software	Vertucci’s classification	Retrospective data from database.	Occurrence of roots, canals and their configuration in first premolar are as follows: 1 root-40.7%, 2 roots- 57.5%, 3 roots-1.7%, 1 canal-3.7%, 2 canals-93.2%, 3 canals- 2.6%, 4 canals-0.4%, type II- 6.8%, type III- 7.7%, type IV- 63.8% and type V-14.8%. Similarly, upper second premolar had 1 root in 88% of teeth, 2 roots in 12%, 1 canal in 38.2%, 2 canals in 61.0%, 3 canals in 3 teeth, then type I in 38.2%, type IIIin 15.3%, type IVin 19.2% and type V in 12.3%.	Frequency of two roots and type IVwas high in upper first premolar while single root and type Iwas predominant in second premolar. Majority of upper premolars had two canals.
[32] Al-Zubaidi, S.M., et al. 2022	1000	Mandibular first and second premolars	Carestream CS 8100 3D CBCT (Carestream Dent LLC, Atlanta, USA)	75 m	CS 3D Imaging Software (Carestream Dent LLC, Atlanta, USA).	Vertucci’s classification	Retrospective data from database.	Study analyzed that 95.5% of first mandibular premolars had single root and two roots were present in 4.5%, 70% of the teeth were accounting for type Ithen type II (14.2%) and type IV was (10.1%). In case of second mandibular premolar 99.2% samples had single root and two roots were found only in four teeth (0.8%). Similarly type I was the highest canal configuration (91.1%) then type II (5.7%)	According to the study incidence of one root and type I canal classification was higher in Saudi sub population although different configurations were present in several roots.
[58] Alfawaz, H., et al. 2019	734	First and second mandibular premolars	CS9300 3D digital imaging system (Carestream, USA) and Planmeca ProMax 3D (PLANMECA, USA)	90–500 μm and ≤ 200 μm	Planmeca Romexis Viewer software (Planmeca, Roselle IL).	Vertucci’sx classification	Retrospective data from database	Type 1 root canal configuration and one root was the prominent finding in mandibular first (96.4%) and second (95.6%) premolar, however type VII was absent in first and type VI & VII were not seen in second premolar. Bilateral symmetry was 93.8%and 97.8% in terms of roots and canal configuration in both the premolars.	Study revealed that one root and canals with type 1 configuration was the prominent finding in mandibular premolars of Saudi population, however premolars with more than one root and varying canal configuration was detected.
[59] Alghamdi, F.T. and W.A. Khalil 2022	2400	Mandibular second premolar	i-CAT 1719 3D digital imaging system (Imaging Sciences International, Hatfield, PA, USA)	90–200 μm	OnDemand 3D Imaging Software (Cybermed, Seoul, South Korea)	Vertucci’s classification	Retrospective data from database.	Study discovered that 98.33% of mandibular second premolar had one root and 1.67% were two rooted, occurrence of two roots on right side was higher in females compared to left side but this was in contrast with male patients. 97.91% of teeth had type I canal configuration, then type II (1.17%) and type IV (0.58%). Significant difference with regard to gender and size was revealed by only type I and type II canal configuration.	The study exhibited that root and canal morphology of second mandibular premolar varies greatly with increased prevalence of one root and one canal and less frequency of two roots.
[60] Mashyakhy, M., et al. 2022	776	Mandibular premolars	3D Accuitomo 170 Morita; Osaka, Japan	0.25 mm	Morita’s i‑Dixel 3D	Vertucci’s classification	Retrospective data from database.	Occurrence of roots, canals and their configuration in the first mandibular premolar are as follows: 1 root-99.5%, 1 canal-69.5%, 2 canals- 29.5%, type I-69.5%, type III-6.3%, type V-22.2% and type VII-0.3%. Likewise, second premolar had: 1 root-100%, 1 canal-96.8%, 2 canals-2.1%, type I-96.8%, type III-1.6% and type V-0.8%.	Study indicated that mandibular first and second premolars had single root and one canal in majority along with few variations.
[61] Alam, F., et al. 2020	1504	Mandibular first and second premolars	Scanora 3D equipment (Soredex, Finland)				Retrospective data from database	Findings in the first premolar: One root-80.85%, two roots-19.14%, one canal-71.27% and two canals-28.72%. Second premolar: One root-88.29%, two roots-11.70%, one canal-71.80% and two canals-28.19%. Statistically significant difference was noted among males and females in case of number of roots of first premolar.	Study stated that variations in number of roots and canal of first and second mandibular premolars is common among males and females of included Saudi population.
[62] Algarni, Y.A., et al. 2021	216	Mandibular first premolar		0.125 mm	CS 3D Imaging Software (Carestream Dental)	Vertucci’s classification	Retrospective data from database	Incidence of canals in females: one canal: 79.2%, two canals: 15.8%, three canals: 5%. Canals evidence in males: one canal: 54.1%, two canals: 33.3%, three canals: 12.5%. Incidence of canals in right premolar: one canal: 72.2%, two canals: 26.2%, three canals: 1%. Canals in left premolar: one canal: 76.7%, two canals: 22.2% and three canals: 2%. Canal configuration in single rooted first premolar: type I:74%, type II:10%, type III;6.5%, type V: 8.1%, and type VI were 1.1%. Similarly in double rooted premolar: types I: 5.5%, type II: 22.2%, type IV: 16.6% and type V were 55.5%.	It can be concluded from the study that mandibular first premolar predominantly had single canal and type I configuration, but variations were found. Hence careful examination is essential.
[63] Al-Shehri, S., et al. 2017	351	Maxillary first molars	I‑CAT (Imaging Science International, Hatfield, PA, USA), Galileos (Sirona Dental Systems, Germany), Carestream CS 9300 (Carestream Health, Inc., Rochester, NY, USA).	0.3 mm	OnDemand3D software (Cybermed, Seoul,Korea)	Vertucci’s classification	Retrospective data from database.	94% of the samples had three separated roots, and 6% fused roots which were higher in females (71.4%). DBRs and PRs were fused more commonly (4.8%) compared to other roots. It was frequent on the right side (61.9%). Occurrence of canals: 2 canals (3.7%), 3 canals (40.4%), 4 canals (55.6%) and 5 canals (0.3%). Type Icanal configuration was major in DBR (99.3%) and PR (100%) whereas MBR had higher frequency of type IV (55.6%).	According to this study majority of maxillary first molars had three roots and four canals. MBR had the fourth canal with prevalence of type IV.
[64] Alamri, H.M., et al. 2020	351	maxillary second molars	Carestream CS 9300 (Carestream Dent LLC, Atlanta, G, USA)	180–300 μm	CS 3D imaging software (Carestream Dent LLC, Atlanta, G, USA)	Vertucci’s classification	Retrospective data from database.	Occurrence of roots: 1 root (0.3%), 2 roots (6.6%), 3 roots (92%) and 4 roots (1.1%). Two canals were significant in females while male patients had higher number of four canals, age and number of canals was inversely proportional.	This study concluded that, three and two rooted maxillary second molars are more common among Saudi population with higher tendency of three roots in males and two roots in females respectively.
[65] Al-Habib, M. and M. Howait 2021	106	Maxillary first molars	iCAT scanner (Imaging Sciences International, Hatfield, PA, USA)	0.2 mm	Vision software (Imaging Science International, Hatfield, PA, USA).	Vertucci’s classification	Retrospective data from database	Study revealed that 92 (86.8%) of maxillary first molars had MB2 canals, 61 (58%) of cases exhibited joining of MB and MB2canals at coronal 14 (23%), middle 17 (27%) and 31 (50%) apical third separately. 2.52 ± 0.76 mm was the mean inter orifice distance connecting two canals at the floor of pulp.	The study concluded that Saudi sub population had higher presence of MB2 canals in maxillary first molars which reduced as the canal reached apical third.
[66] Al-Fouzan, K.S., et al. 2013	470	Maxillary first and second molar					Prospective study	It was evident that included maxillary 1st and 2nd molars had two canals in the mesial root. 33.1% of 1st molars end in one foramen which were confluent type, and two separate foramina were noted in 18.2%. Similarly, 2nd molar had 13.6% of one foramen for two canals and 6.2% of two foramens.	Study concluded the higher incidence of two canals in 1st maxillary molar and evidence of mesio palatal canal in the 1st and 2nd maxillary molars.
[67] Mashyakhy, M., et al. 2022	624	Maxillary molars	3D Accuitomo 170, MORITA, Japan	0.25 mm	Morita’s i‑Dixel 3D software	Vertucci’s classification	Retrospective data from database	All upper 1st molar had three separated roots, four canals were evident in 80% and three canals in 14.2% teeth. Canal configuration in MB root was type I followed by type II, IV, III, V and type I was 100% in distobuccal and palatal roots. 99.3% of upper second molar had three separated roots and 2% were extra palatal root and 66.4% had four canals while 33.6% were three canals. Type I was 100% in palatal and distobuccal roots.	Maxillary molars had greater variations with higher frequency of four canals and type I configuration.
[68] Mirza, M.B., et al. 2022	86	Maxillary molars	Carestream CS9300 (Carestream Dent LLC, Atlanta, GA, USA) and Kavo OP3D Pro (KaVo Imaging, Hatfield, PA, USA)	180-300 mm		Vertucci’s classification and new system of classification	Retrospective data from database	Three roots were noted in 100% max 1st molars while second molar had three roots in 95.5%, two roots (2.7%), four and one root in 0.75% and five roots in 0.3%. type I, II, III and IV vertucci’s type were seen in 1st molar and 2nd molar had type I, II and IV. MB root of max molars had type I, II, III and IV configuration.	Predominantly 1st maxillary molar had single root while 2nd molar had varied root numbers and both the molars displayed variations in terms of canals and their configuration. Both genders showed statistically significant difference.
[69] Syed, G.A., et al. 2022	400	Maxillary molars				Vertucci’s classification	Retrospective data from database	Mesio buccal root of 1st and 2nd molar had type I (20.25%), type II (17.25%) and type III (17.25%). Right and left 2nd molars had higher frequency of type I and type II was major in first molar.	Study concluded higher presence of mesio buccal root with different configuration.
[70] Aldosimani, M.A., et al. 2021	1377	Mandibular first and second molars	Planmeca Promax 3D Max digital imaging device (Planmeca, Helsinki, Finland)	0.1-0.4 mm	Planmeca Romexis 5.2 (Planmeca, Helsinki, Finland).	Pomeranz et al. classification	Retrospective data from database.	Study discovered 12 (0.9%) mid-mesial canals from the population, 1.3% in first mandibular molar and 0.4% in second mandibular molar. Confluent type of mid-mesial canal was more common (*n* = 10).	Study discovered that confluent type was more common MMC configuration then fin type, and independent type was not found
[71] Alfawaz, H., et al. 2019	1210	Mandibular first and second molars	Planmeca Promax 3D max (Planmeca, Finland) and Cs 9300 (Carestream Health, USA)	200 and 300 μm	Romexis data viewer software (Romexis, Finland)	Fan et al. criteria	Retrospective data from database	C shaped canal system was present in one first molar (0.19%) whereas 62 (9.1%) in second molars (9.1%); majority of C shaped root anatomy was unilateral (53.85%). Females had higher chances of c shaped root canal system than males, longitudinal grooves were more commonly present on the lingual surface of the root (58.1%)	The researcher discovered that Saudi population in the study had one C shaped canal in mandibular first molar (0.19%) and 62 in second molars (9.1%). C shaped anatomy was more frequent in females than males and unilateral C shapes canals were higher than bilateral. Occurrence of longitudinal groove was higher on the lingual surface of roots.
[72] Alnassar, F., et al. 2022	145	Mandibular first and second molars	Planmeca ProMax 3D (PLANMECA, Roselle, IL, USA).	90–300 μm		Pomeranz et al. classification	Retrospective data from database	Study discovered that out of all the teeth only one extra MD canal was found in first mandibular molar (0.7%) which was confluent with the DL canal whereas mandibular second molar had no extra MD canal. Further the distance from MB and DB canal was 1.9 mm, MD and DL canal- 1.4 mm and from CEJ to MD canal was 3.1 mm respectively.	Study noted that the occurrence of MD canal was remarkably low in the studied population.
[73] Mashyakhy, M., et al. 2019	174	Mandibular first molars	3D Accuitomo 170 (MORITA, Japan)	0.25 mm	Morita’s i‑Dixel 3D imaging software	Vertucci’s classification	Retrospective data from database.	Of the studied lower first molars, 97.1% were double rooted and 3 roots were in 2.9%. With reference to canals: Two canals- 1.7%, Three canals-73% and Four canasl-025.3%. In case of four canals, 90.9% extra canals were in distal root while distolingual root had 9.1%. Type I (77%) and type IV (64.9%) canal configuration was the most common in distal and mesial roots.	Canal configurations of mandibular first molar varied greatly in the studied Saudi population. Occurrence of three roots was low while three canals were common finding. Varying degree of bilateral symmetry was presented by number of canals and their configurations.
[74] Mashyakhy, M., et al. 2021	657	First and second mandibular molars	3D Accuitomo 170 (MORITA, Japan)	0.25 mm	Morita’s i‑Dixel 3D imaging software	Vertucci’s classification	Retrospective data from database.	First mandibular molars had 2 roots- 95.4%, 3 canals-64.5% and type IV- 57.9% in mesial roots. While second molars had 2 roots-89.6%, 3 canals-80.4% and type IV-39.4%. Either side of premolars showed identical anatomy.	It could be revealed from the study that majority of both the molars were double rooted and had three canals. Type IVwas major canal configuration in mesial canals of both the molars while type Iwas major in distal canal.
[75] Al-Alawi, H., et al. 2020	741	Mandibular first molars	Promax 3D Max (Planmeca, Helsinki, Finland), Galileos Comfort (Sirona Dental Systems GmbH, Bensheim, Germany) and CS9300 (Carestream Dental LLC, Atlanta, GA, USA)	0.2-0.4 and 0.9 mm	Planmeca Romexis (version 5.2; Planmeca)	Carlsen and Alexandersen, Song et al. classification	Retrospective data from database	Study revealed the presence of supernumerary roots (4.5%) among which 4.2% were on distolingual side and 0.35 were mesiobuccally. They usually had single canal occurring unilaterally. However bilateral occurrence was 1.3%.	Study concluded that the incidence of supernumerary root among the Saudi population was 4.5%.
[30] Iqbal, A., et al. 2022	3420	Maxillary and mandibular anterior	3Dx SCANORA (Tuusula, Finland, Nahkelantie 160)	0.25 mm	On-demand 3D version software 1.0.10.6388 (Daejeon, Korea, Yuseonggu)	Vertucci’s classification and new system of classification	Retrospective data from database	It was evident from the study that major type of configuration was type I and 1TN1 in maxillary anterior while predominant type was type I in lower anterior then type III and type IV. According to Ahmed’s classification majority was 1TN1 then 1TN1-2-1 and 1TN1-2. Root canals of lower canine varied greatly in females then males. Ethnicity and age had greater impact on the canal variation of upper laterals and lower anterior.	Lower anterior displayed broad canal variation and complexity in the root canal morphology. Differences in relation to root morphology was not greatly determined among genders except for the canine.
[76] Mashyakhy, M 2019	794	Maxillary and mandibular canines	3D Accuitomo 170 (MORITA, Japan)	0.250 mm	Morita’s i-Dixel 3D imaging software	Vertucci’s classification	Retrospective data from database.	Mandibular canines were analyzed with two roots- 2.7%, two canals- 9.3%, type III- 6.1% and type V- 3.2% Vertucci’s classification. No two roots were found in maxillary canines and type IIIVertucci’s configuration was seen only in 1%. Further roots and canals presented bilateral symmetry in both the canines.	Study stated that both the sides of canals conveyed bilateral symmetry in mandibular canines.
[77] Mirza, M.B 2022	1880	Maxillary and mandibular canines	Carestream CS9300 (Carestream Dent LLC, Atlanta, GA, USA)	180-300 mm	Not mentioned	New classification and Vertucci’s classification	Retrospective data from database.	One root was in 100% of maxillary canine while mandibular canine had 98.7% one root, 1.3% two roots, 97.48% type I,0.21% type II, 1.055 type III and 1.26% type V Vertucci’s classification. They represented significant difference in relation to canal morphology and number of roots as described by Vertucci but no such differences were observed by Ahmed at al.	Study concluded that mandibular canine indicated greater variation in relation to number of roots and morphology of canals however, one root and type Iwas predominant.
[78] Almohaimede, A.A., et al. 2021	1328	Maxillary and mandibular canine	Planmeca ProMax 3D (PLANMECA, Roselle, IL, USA) and CS9300 3D digital imaging system (Carestream, Rochester, NY)	75–600 μm	Planmeca Romexis Viewer software (Planmeca, Roselle IL)	Vertucci’s classification	Retrospective data from database	All the maxillary canines were single rooted (100%), one canal in 98.1% and two canals in 1.89%. However, mandibular canine had 97.11% one root, two roots (2.88%), one canal (90.05%) and 9.94% were two canals. Females had higher incidence of two roots than male. Type I configuration was common (94.9%) followed by type V (1.8%), type III (1.7%), type II (1.1%), type IV (0.4%) and type VII (0.2%).	Study concluded that incidence of one root, single canal and type I configuration was higher in maxillary and mandibular canine. Although mandibular canines had more variations compare to maxillary canine.
[79] Alshammari, S.M., et al. 2023	286	Maxillary and mandibular premolar	SCANORA 3Dx (Nahkelantie 160, Tuusula Finland)	0.25 mm		Vertucci’s classification and Ahmed’s classification	Retrospective data from database	Incidence of one root: 73% (max right 2nd premolar), 70% (max left 2nd premolar), 53% (max right 1st premolar), 38% (max left 1st premolar). Evidence of two roots: 62% (max left premolars), 47% (max right premolars). Whereas mandibular premolars frequently had single root. Frequency of two canals: 82% (max left 1st premolar), 76% (max left 2nd premolar), 74% (max right 1st premolar), 58% (max right 2nd premolar). 18–29% (mand premolars). Max premolar had vertucci’s type IV, type I in mand premolars however 2% of type III was noted in mand left 1st premolar. (2 FP B1 P1) was predominant in ma 1st premolar (73% and 81%) then (1 FP 2) 19%.	Among the investigated population frequency of second canal was less in maxillary and mandibular premolars compared to one root and single canal.
[80] Boreak, N.M., et al. 2022	1666	Maxillary and mandibular premolars	3D Accuitomo 170 (MORITA Japan)	0.25 mm	Morita’s i‑Dixel 3D		Retrospective data from database	Of the studies premolars, majority of first and second right and left maxillary premolars had two canals (93%, 95%,57%,43%) while max right 2nd premolar had higher number of one canal compared to other. All the mandibular premolars had single root and canal. Circular cross-sectional shape was major among 1st and 2nd max premolars at coronal, middle and apical area than flattened and oval type with exception of right max 2nd premolar. Similarly common cross section in coronal area of 1st and second mand premolar was oval flattened and circular.	Study focused on the cross-sectional canal shape of maxillary and mandibular premolar indicating that circular shape was prominent in the apical area while variations were noted in the coronal and middle area.
[81] Iqbal, A., et al. 2022	1443	Maxillary and mandibular premolars	SCANORA 3D CBCT unit (Soredex Inc., Tusula, Finland)		On Demand 3D software	Vertucci’s classification	Retrospective data from database	Maxillary 1st premolar had two roots (52.6%) then one root (26.5%), two fused roots in 19.3%, two separated roots in 0.8% and three roots were 0.5%. Type I was major followed by others. Likewise single root with one canal and type I was common finding in maxillary 2nd premolar. Mandibular 1st and 2nd premolar predominantly had one root and single canal with type I and absence of three roots.	It was concluded from the study that wide variations of root canal morphology were noted among upper and lower premolars.
[82] Alhujhuj, R.R., et al. 2022	123	Maxillary and mandibular first molars	I-CAT Vision QTM (Imaging Sciences International Hatfield, PA, USA. Version 1.9.3.14	0.25 mm	BlueSkyPlan (Version 4.7.55, GmbH, Langenhagen, Germany)	Vertucci’s classification	Retrospective data from database	Findings of the study are as follows: Right maxillary 1st molar: three roots (90.2%), four roots (9.8%), three canals (78.9%), four canals (21.1%), MB root had type I (87%), type IV (9.8%), type II (3.3%). Palatal root had 99.2% type I. MB root of left molar: 84.6% (type I), 9.8% (type IV), 5.7% (type II). Left mandibular first molar: three roots (85.4%), four roots (12.2%), three canals (73.2%), four canals (25.2%), type I (100%). Right mandibular first molar: three roots (85.4%), four roots (13.0%), two roots (1.6%). Three canals (74.0%), four canals (25.2%) and two canals (0.8%). Type I configuration was 100%.	Study concluded that the majority of studied Saudi population had three roots and three canals along with type I.
[83] Mashyakhy, M., et al. 2020	1433	Mandibular premolars and molars	3D Accuitomo 170 (MORITA, Japan)	0.25 mm	Morita’s i‑Dixel 3D imaging software		Retrospective data from database.	Incidence of C-shaped canals was 1.5% in lower first premolar, second premolar-0.80%, second molars-7.9% and absent in first molars. Different types of C-shaped canals were found in both molars and premolars while C2 was prominent in premolars and C3 was in second molars. External longitudinal grooves were dominant on mesiolingual surface of premolars and lingually in molars. Increased incidence of C-shaped canals was seen in second molars among females but premolars had no difference.	Occurrence of C-shaped canals was higher in second mandibular molars but less in lower premolars. There was no significant difference in relation to gender and sides.
[84] Mashyakhy, M. and A.S. Abu-Melha, 2021	5223	Permanent maxillary and mandibular teeth	3D Accuitomo 170 CBCT unit (J Morita, Kyoto, Japan)	0.25 mm	i-Dixel 3D imaging suite	Vertucci’s classification	Retrospective data from database.	Roots displayed 100% bilateral symmetry in the maxillary arch except first premolar while higher variations were seen in lower arch except second premolar. Canals and their configuration of upper central and lateral incisors had 100% bilateral symmetry while asymmetry was noticed among second molars.	Maxillary arch was more symmetrical than the mandibular arch. Evidence of bilateral symmetry was highest in terms of roots than canals and the least was presented by canal configuration.
[85] Mashyakhy, M. Mashyakhy, M., et al. and G. Gambarini 2019	5254	Permanent maxillary and mandibular teeth	3D Accuitomo 170 (MORITA, Japan)	0.25 mm	Morita’s i‑Dixel 3D imaging software	Vertucci’s classification	Retrospective data from database	No remarkable difference was noted among males and females in terms of number of roots, while significant difference was shown by canals of upper teeth among both the genders. Canal configuration was statistically significant in upper roots between males and females. Canal configuration of anterior and premolar of lower arch were significantly different but no difference was found in mesial roots of both the molars however, it was opposite in case of distal roots.	Study concluded that number of roots in males and females of Saudi population were not significantly different but the canals and their configuration had differences in upper and lower teeth between both the genders

**Table 3:** Characteristics of the included CBCT in-vitro studies

Study Reference	Sample Size	Sample type	CBCT machine	Voxel size (Resolution)	Software used	Classification System	Technique	Results	Conclusion
[86] Maghfuri, S., et al. 2019	100	Maxillary first premolars	3D Accuitomo170	12,500 μm	I-Dixel imaging software	Vertucci’s classification	Extracted premolars were washed in under and placed in sodium hypochlorite (2.5%) then teeth were mounted in artificial jaw and CBCT was done.	Occurrence of root is as follows: one root (36%), two roots (61%) and three roots (3%). Evidence of canals: two canals (97%), three canals (3%) and one canal was not found. Incidence of canal configuration: Type IV (75%), Type V (13%), Type II (7%), Type VIII (3%) and Type VI (2%).	It was displayed from the study that maxillary first premolar of involved Saudi population had higher incidence of two roots, two canals and type IV root canal configuration
[87] Chourasia, H., et al. 2017	100	Mandibular first premolar				Vertucci’s classification	Extracted teeth were washed and stored in 10% formalin followed by access cavity preparation and dissolution of pulpal tissues in sodium hypochlorite (5.25%) then clearing, Decalcification by nitric acid (5%) and dehydration then transparent teeth were analyzed under dental microscope.	Study revealed that 80% of mandibular 1st premolar had one root, 18% were double rooted and 2% had three roots. One canal was found in 72%, 26% were two canals and 2% were three canals. One apical foramen was noted in 73%, two foramens were in 19%, three foramens were found in 2% and evidence of multiple apical foramina was seen in 6%. Occurrence of canal configuration: type I (69%), type III (8%), type IV (4%), type V (16%). 38% of teeth had lateral canals and 16% showed internal communication.	Despite of the fact that majority of 1st mandibular premolars were single rooted with one canal, double roots and two canals were also found indicating morphological variations among Saudi population.
[88] Alrahabi, M. and M.S. Zafar 2015	100	Maxillary first molars		76 μm	Imaging software (CS 3D; Care stream Health, Inc., 2011)	Vertucci’s classification	Study included 100 maxillary first molars which were extracted freshly, 5% NaOCl was used to clean the teeth followed by washing with ultrapure water then teeth were place in normal saline and further radiographic evaluation was carried out.	Three distinct roots were present in 94% of maxillary first molars and 6% of molars were identified with four roots. Single root canal with type 1 vertucci’s classification was noticed in distobuccal and palatal roots whereas MB root with one root canal (29.6%) and two canals (70.6%) were found. Type II (47%) configuration was highest in MB roots then type I (29.4%) followed by type IIIand IV (11.8%) individually.	Study stated that first maxillary molars had higher incidence of three separated roots and presence of fused roots was rare. Type Ivertucci canal configuration was significantly higher in DB and palatal roots whereas, type Ior IIconfiguration was more common in MB roots.

**Table 4:** Characteristics of the included Micro- CT studies

Study Reference	Sample Size	Sample type	Micro-CT machine	Pixel size (Resolution)	Software used	Classification System	Technique	Results	Conclusion
[89] Elnour, M., A. Khabeer, and E. AlShwaimi 2016	100	Maxillary second premolars	SkyScan 1172 X-ray scanner (Bruker Corp., Antwerp, Belgium)	27.4 μm	SkyScan CT-Volume v2.2 (Bruker Corp., Antwerp, Belgium)	Vertucci’s classification	100 s maxillary premolars were stored in 10% formalin followed by washing under water to remove the debris and then microscopic and radiographic evaluation was done.	Study revealed the following findings: one root-67%, two roots-30%, three roots-3%, one canal-30%, two canals-65%, three canals-5%, type I-17%, type II-7%, type III-9%, type and V- 23% and type VII-2%. Detailed description of canal orifice, apical foramen and apical delta was recorded which is as follows. One canal orifice: 55% Two canal orifices: 45% One apical foramen:34% Two apical foramina:50% Three apical foramina:11% Four apical foramina:4% Five apical foramina:1% In addition to this accessory canals were found in 8% of teeth, intercanal communication was also noticed in 8% of second premolar at different locations and isthmuses were in middle (1%) and coronal third (1%).	Study concluded that Saudi subpopulation had complex root anatomy of second maxillary premolars.
[90] El Ashiry, E.A. and N.M. Farsi 2018	100	Maxillary first molars	SkyScan micro-CT scanner (SkyScan 1172, SkyScan, Bruker, Belgium)	13.4 μm	(SkyScan 1172, SkyScan, Bruker, Belgium)		Disinfection of all the molars was done by sodium hypochlorite (5.25%), then placed in normal saline for 24 h then micro-CT was performed.	Incidence of pulpal canal orifice: 47.37% (4), 35.09% (5), 10.53% (6) and 7.01% (3).	Study concluded that increased incidence of 4 and 5 pulpal canal orifice then 3 and 6 canal orifices.
[91] Alfouzan, K., et al. 2019	65	Maxillary first and second molars	SkyScan 1172 (Bruker microCT, Kontich, Belgium)	13.6 μm	CTAn software (Bruker microCT)	Pomeranz et al. classification	Extracted teeth were cleaned and stored in normal saline.	Among the included samples 80% of first molars had two MB canals and 17% had three. While 80% and 13% of second molar had two and three MB canals. Presence of MB2 canal at different level is as follows: Chamber floor: 70% (1st molar), 61% (2nd molar). 1 mm depth: 15% (1st molar), 18% (2nd molar). 2 mm depth: 3% (1st molar), 18% (2nd molar). Deeper than 2 mm: 4 (1st molar), 1 (2nd molar). Confluent canal type was dominant then independent and fin type.	70% and 61% of MB2 canal could be easily found in 1st and 2nd maxillary molars among included Saudi population and the remaining MB2 canals were detected at deeper level.

## Specifications

The present systematic review included the studies conducted in the different regions of Saudi Arabia. It is evident from the literature that differences in ethnicity contributes to the variations in root canal anatomy [44]. The results of the present study indicate that most of the anatomical features of the teeth were similar in Saudi Arabian population except for few studies where Saudi Arabian-sub regions had variations. Clinically, the population associated variations would help the clinician in understanding the anatomy properly and planning the treatment accordingly by avoiding any possible damage during the procedure.

Sample size calculation is essential for any research as smaller size of samples can mislead the findings and causes greater variations in results. Numerous factors affect the sample size calculation such as study type, population, cost, research questions and objective [45]. In the following systematic review, as many as 93.61 of the studies did not mention the method of sample size calculation which could be a weak point and can possibly alter the results.

Calibration is another important factor for attaining definitive outcomes. Calibrating is crucial to reduce the errors and minimize the potential bias, hence the investigators must be experienced and desirably calibrated to judge the clinical results and observations [46]. Cohen’s Kappa test is preferably used for calibration. In the present review, 36.17% of the studies did not mention calibration, since root canal anatomy is an important topic, the researcher must be calibrated to evaluate the methodology (Table 5).

**Table 5:** Specifications of the studies

S.no	Study reference	Region	Ethical approval	Sample size	Method of sample size calculation	Calibration
1	[47] Ghabbani, H.M., A.A. Marghalani, and H.R. Alabiri 2021	Al‑Madinah Al‑Munawara	Yes	1624	Not mentioned	Not reported
2	[48] Mashyakhy, M 2019	Jazan city	Yes	822	Not mentioned	Reported
3	[49] Mohamed, A.N., et al. 2021	Qassim region	Yes	376	Not mentioned	Reported
4	[50] Alaboodi, R.A., S. Srivastava, and M.Q. Javed 2022	Qassim region	Yes	928		Reported
5	[51] Al-Dahman, Y., et al. 2019	Riyadh	Yes	454	Not mentioned	Not reported
6	[52] Almohaimede, A., et al. 2022	Riyadh	Yes	1370	Not mentioned	Reported
7	[53] Alobaid, M.A., et al. 2022	Saudi-sub population	Yes	1260	Not mentioned	Reported
8	[54] Alshayban, M., et al. 2022	Riyadh	Yes	1769	Not mentioned	Reported
9	[55] Alqedairi, A., et al. 2018	Riyadh	No	652	Not mentioned	Reported
10	[56] Al‑Zubaidi, S.M., et al. 2021	Hail city	Yes	1000	Not mentioned	Reported
11	[57] Mashyakhy, M 2021	Jazan city	Yes	710	Not mentioned	Reported
12	[86] Maghfuri, S., et al. 2019	Southern region of Saudi Arabia	No	100	Not mentioned	Not Reported
13	[32] Al-Zubaidi, S.M., et al. 2022	Hail city	Yes	1000	Not mentioned	Not reported
14	[89] Elnour, M., A. Khabeer, and E. AlShwaimi 2016	Easter province of Saudi Arabia	Yes	100	Not mentioned	Not reported
15	[58] Alfawaz, H., et al. 2019	Riyadh	Yes	734	Not mentioned	Reported
16	[59] Alghamdi, F.T. and W.A. Khalil 2022	Jeddah	Yes	2400	Mentioned	Reported
17	[60] Mashyakhy, M., et al. 2022	Jazan city	Yes	776	Not mentioned	Reported
18	[61] Alam, F., et al. 2020	Sakakah	Yes	1504	Not mentioned	Not Reported
19	[62] Algarni, Y.A., et al. 2021	Aseer region	Yes	216	Not mentioned	Reported
20	[87] Chourasia, H., et al. 2017	Southern region of Saudi Arabia	No	100	Not mentioned	Not Reported
21	[63] Al-Shehri, S., et al. 2017	Riyadh & Dammam	Yes	351	Not mentioned	Reported
22	[64] Alamri, H.M., et al. 2020	Al-kharj	No	351	Not mentioned	Not Reported
23	[65] Al-Habib, M. and M. Howait 2021	Jeddah	Yes	106	Not mentioned	Reported
24	[88] Alrahabi, M. and M.S. Zafar 2015	Al‑Madinah Al‑Munawara	Yes	100	Not mentioned	Not Reported
25	[66] Al-Fouzan, K.S., et al. 2013	Riyadh	No	470	Not mentioned	Not Reported
26	[67] Mashyakhy, M., et al. 2022	Jazan city	Yes	624	Not mentioned	Reported
27	[68] Mirza, M.B., et al. 2022	Al-Kharj	Yes	86	Not mentioned	Reported
28	[69] Syed, G.A., et al. 2022	Jeddah	Yes	400	Not mentioned	Not reported
29	[90] El Ashiry, E.A. and N.M. Farsi 2018	Jeddah	Yes	100	Not mentioned	Reported
30	[91] Alfouzan, K., et al. 2019	Riyadh	Yes	65	Not mentioned	Not reported
31	[70] Aldosimani, M.A., et al. 2021	Riyadh	Yes	1377	Not mentioned	Not reported
32	[71] Alfawaz, H., et al. 2019	Riyadh	No	1210	Not mentioned	Reported
33	[72] Alnassar, F., et al. 2022	Riyadh	Yes	145	Not mentioned	Reported
34	[73] Mashyakhy, M., et al. 2019	Southern region of Saudi Arabia	No	174	Not mentioned	Reported
35	[74] Mashyakhy, M., et al. 2021	Jazan city	Yes	657	Not mentioned	Reported
36	[75] Al-Alawi, H., et al. 2020	Riyadh and Al-kharj	Yes	741	Not mentioned	Reported
37	[30] Iqbal, A., et al. 2022	Sakakah	Yes	3420	Mentioned (G power 3.1.9.4 software)	Reported
38	[76] Mashyakhy, M 2019	Jazan city	Yes	794	Not mentioned	Reported
39	[77] Mirza, M.B 2022	Al-kharj	Yes	1880	Not mentioned	Reported
40	[78] Almohaimede, A.A., et al. 2021	Riyadh	Yes	1328	Not mentioned	Reported
41	[79] Alshammari, S.M., et al. 2023	Sakakah	Yes	286	Not mentioned	Not reported
42	[80] Boreak, N.M., et al. 2022	Jazan city	yes	1666	Not mentioned	Not reported
43	[81] Iqbal, A., et al. 2022	Sakakah	Yes	1443	Mentioned	Reported
44	[82] Alhujhuj, R.R., et al. 2022	Al-Asha	Yes	123	Not mentioned	Not reported
45	[83] Mashyakhy, M., et al. 2020	Jazan city	Yes	1433	Not mentioned	Reported
46	[84] Mashyakhy, M. and A.S. Abu-Melha, 2021	Jazan city	Yes	5223	Not mentioned	Reported
47	[85] Mashyakhy, M. Mashyakhy, M., et al. and G. Gambarini 2019	Jazan city	No	5254	Not mentioned	Reported

## Discussion

The present systematic review assessed the frequency of roots and root canal configuration in the permanent human teeth in the different regions of Saudi Arabian population. Both clinical and *in-vitro* data from various CBCT and micro-CT studies which provides information of morphological variations of teeth anatomy amongst Saudi population were included in the present review. The findings of this systematic review revealed significant variations in root canal morphology among different regions of the Saudi Arabian population. These variations can be attributed to a combination of factors, including genetic diversity, environmental influences, and regional differences in dietary habits and oral hygiene practices [5]. Additionally, variations may arise from differences in sample size, examination techniques, and the experience of examiners [49, 52].

It is important to note that root canal anatomy is inherently complex, and these variations emphasize the need for clinicians to approach endodontic procedures with a comprehensive understanding of the potential anatomical variations that may be encountered [92]. Failure to do so can result in missed canals, inadequate cleaning and shaping, and ultimately, treatment failure [93]. The risk of bias assessment, based on modified version of earlier risk of bias assessment tool was conducted for the included studies [37]. This assessment is essential to evaluate the methodological quality of the studies and consider potential sources of bias. It included five objectives to assess the studies, they were (1) samples size calculation where, studies were evaluated to know if proper tool is used to calculate the sample size, (2) Reporting and quality of data by determining various factors like voxel size, x-ray machine and software used, (3) Clear description of results, (4) Reliability of an observer which is an essential criteria to avoid human error and minimize the potential bias and (5) Attrition bias where sample loss is reported by proper sampling method used in the study to cover the mentioned population in a defined region. It mainly features the population in each region like Riyadh rather than generalizing it (eastern province of Saudi Arabia). Based on the above-mentioned objectives, the studies were classified as low, moderate, and high risk of bias (Figs. 3,1 and 5).

Sample size calculation is important in any research to generalize the results and obtain a justifiable conclusion. Sample size calculator and G* POWER software are usually used for that purpose. Sample size calculations was mentioned only in three included studies in this systematic review. In the case of observational studies, especially those involving anatomical evaluations, sample size calculations may not be applicable in the same way as in clinical trials or experimental studies [46]. Sample size calculations are a crucial component of research design, primarily in experimental studies, to ensure adequate statistical power and the ability to detect meaningful effects. However, not conducting the sample size calculation or not reporting it weakens the study [94].

Similarly, calibration and quality of image are important for accurate results. Calibration confirms accurate measurements, while image quality affects the visibility of anatomical details. These factors are primarily important in studies where root canal anatomy is explored. Calibration establishes accuracy. Good quality of images is necessary for precise root canal analysis. Poor quality images provide unclear details and lead to inaccurate conclusions. Valid calibration and high-quality images enhance reliability [46].

Acknowledging the importance of radiography in endodontics for diagnosing and guiding the entire procedure, CBCT is regarded as the standard of care [95–97]. The scanner of CBCT is simple, cost effective and less complicated, constructing 3D images which appreciates the tooth anatomy and their surrounding structures along with varied anatomy and teeth anomalies [98–100]. It requires lesser radiation exposure and minimum period of time to produce the images [101]. However, CBCT has limitations in terms of voxel size and resolution, which may affect the ability to detect fine anatomical details [93] and because the teeth are encased in bone with surrounding structures that may cause artifacts [102].

Micro-CT is another 3D imaging technique with higher resolution used to provide qualitative and quantitative analysis of the root and canal anatomy [103]. It is non-destructive, non-invasive and reproducible method which gives detailed information of fine anatomical variations including the root apex morphology [104, 105]. As it is non-destructive method of imaging, it allows scanning of the same samples thus allowing examination of the volumetric features of the root canals after intervention (such as comparing shaping properties of certain endodontic file systems) and distinctively, the same data can be further used for mechanical testing [23]. It is also applied in tissue engineering and biomechanics. It determines the stability of implants and mineral consideration of teeth [106, 107]. Micro-CT is typically used in research settings due to its higher cost and the need for specialized equipment [22]. In studies that utilized micro-CT, researchers were able to examine root canal morphology at a finer scale, often revealing accessory canals, inter-canal communications, intricate canal configurations and apical foramen anatomy [89].

The micro-CT studies included in this review provided detailed information on the root canal morphology [89–91]. A study conducted in Easter province of Saudi Arabia, evaluated the root morphology and canal system of maxillary second premolars [89]. According to this micro-CT study, single root (67%) and two canals (65%) was predominant finding along with complex root canal configuration where, type IV and V were found in majority of teeth (23%) followed by other type of configuration. In addition to the above findings, micro-CT provided other detailed descriptions of the apical foramen, apical delta, accessory canals, intercanal communications and isthmuses of maxillary second premolars as shown in Table 4.

Another micro-CT study included in this systematic review was conducted on maxillary first molars to detect extra root canal orifices which is usually difficult due to dentin ledge that can cover the floor of pulp chamber [90]. Commonly it is approved that maxillary first molars have three or four pulp canals, but using 3D images by micro-CT this study evaluated the presence of three (7.01%), four (47.37%), five (35.09%) and six (10.53%) pulp canal orifices [90].

Similarly, one more micro-CT study was carried on maxillary molars [91]. They are considered to have the most intricate root canal morphology than all maxillary teeth, because of the presence of second mesiobuccal canal in the mesiobuccal root which highly varies in its location. Higher failure rate of treatment is associated with failure to locate MB2 canals [108, 109]. As micro-CT produces images of higher resolution, this study evaluated the presence of MB and MB2 canals in maxillary molars (Table 4). Such micro-CT studies offer a complementary perspective to CBCT and underscore the importance of utilizing advanced imaging techniques for comprehensive anatomical assessments.

The anatomy of the root is complex which differs between the teeth as well in the same tooth among different individuals. Root length is not uniform, it may divide and rejoin at varying points and may possess extra canals or irregularities [110]. Therefore, through knowledge of normal and possible variation of the root is essential to successfully perform the debridement, biomechanical preparation and obturation of canals, as lack of understanding and untreated roots adversely affects the outcome and prognosis of the treatment [111].

Results of this systematic review showed that maxillary central and lateral incisors had 100% single root and canal with Vertucci’s type I configuration in the studies conducted among the population of Jazan city [84, 85]. While another study conducted in the Sakakah region, noticed variations in the canal configuration, where majority of maxillary central and lateral incisors had Vertucci’s type I and ^1^TN^1,^ however 12 out of 570 samples had type III and ^1^TN^1–2−1^ [30]. This result could be due to differences in the region.

Canines are the longest teeth in the mouth and they are aesthetically important because of their position in the mouth and functionally essential due to their part in the development of occlusion [77]. It was found that maxillary canine had 100% single root while some variations were detected in the canals and their configuration like single canal was in the range of 98–99% among the studies conducted in the Jazan city and Al-Kharj along with Vertucci’s type I being the predominant (97-100%) followed by type III (1-2%) [76, 77, 84, 85]. However, one study conducted in the Riyadh presented different results where, type I was 97.94% followed by type V (1.1%) and type II and III were 0.47% respectively [78]. This inconsistency in the outcome could be attributed to various factors such as regional differences, sample size and specifications of the evaluation tools [77]. Saudi Arabia is a diverse country with a significant expatriate population, and this diversity can have implications for the observed variations in root canal anatomy. The included studies primarily focused on the Saudi Arabian population, but it is important to acknowledge that this population is not homogenous. Different regions within Saudi Arabia may have distinct ethnic compositions due to historical migration patterns [30].

The majority of mandibular incisors have single root and canal, but certain percentage of variations may be present such as second root canal, lateral root canal and apical delta [48]. Among the included studies of different regions in the present systematic review, 100% of central and lateral incisors were single rooted [47, 49, 50, 53, 54]. However, a very minor percentage of two roots variant was found in lateral incisors in Jazan city (0.5%,1%) [48, 84] and 0.1% in Riyadh [52]. Frequency of one canal and type I was higher in both the incisors. Evidence of two canals in the central incisor was 26.3-55.6% in the regions including Jazan, Qassim region and Riyadh [48–50, 52–54, 84, 85]. While lateral incisors had two canals in the range of 30.8-55.6% [48–50, 52, 54, 84, 85]. Higher frequency of two canals was noticed in the Qassim region and Riyadh (55.6% & 43%) [49, 52]. A worldwide prevalence study conducted by Martin et al., investigated the occasion of a second lingual canal in mandibular incisors in different countries, including Saudi Arabia, found the second canals in 34.7% and 33% of mandibular central and lateral incisors, respectively. According to this study, the presence of lingual canals in mandibular incisors is more in Asian, European and middle East countries than African and American countries. These variations can be attributed to different ethnicity, races, genetic differences or adaptation to specific environment, jaw size and migratory process [112]. Type I configuration was higher (47.3-80.2%) followed by type III (13.6-45.7%) in the central incisors [47, 48, 50, 52–54]. A study conducted by Mohamed, A.N., et al., the Qassim region displayed higher type III configuration (22.9%) than type I (20.5%) [49]. Similarly, higher root canal configuration in the lateral incisors was type I (23.9-69.2%) followed by type III (22.1%42.5%) [47–50, 52, 54, 84, 85]. Alterations in the results as mentioned above could be due to differences in the sample size, regions, and experience of the examiners [49, 52].

Mandibular canines are generally single rooted with one canal, however, variations existed. Based on the literature 15% of the canines are reported to have single root with two canals and 5% of them were recorded with two roots and two canals [51]. Results of the included studies in this review which were conducted in different regions like Riyadh, Jazan and Al-Kharj revealed higher incidence of single rooted mandibular canine (95.1-99%) with slight percentage of two rooted teeth (0.2-2.7%) [51, 54, 76–78, 84, 85]. Single canal was predominant (90.75–93.4%) than two canals (75 − 9.3%) [76, 84, 85]. Likewise type I configuration was higher (86.1%-97.48) than type III (1.05-6.1%) followed by other types [51, 54, 76–78, 84, 85].

Maxillary premolars are one challenging tooth type to treat endodontically due to variations in the root, canal and their configurations [55, 56]. Maxillary first premolars among the included studies performed in the different regions including Riyadh, Hail city, Jazan, Sakakah and Southern region of Saudi Arabia, predominantly had higher percentages of two roots (51.2-75.1%) compared to single root (23.7-40.7%). Three roots were also found in small percentage (0.5-3%) but they were present in males and absent in females [55–57, 81, 84–86].

Two canals were more frequent (74.82-97%) than one (3.7-7.8%) and three canals (1.6-3%) [56, 57, 79, 84–86]. Four canals were present in (0.3-0.6%) of first premolar in the Jazan city [57, 85], in contrast to this, one study conducted in the southern region of Saudi Arabia had no presence of single canal [86]. Type IV canal configuration was higher (57.8-75%) than other types [55–57, 84–86] except one study performed in Sakaka region where type I configuration was higher (58%) [81]. Variations in the results could be due to sample size, examination technique and regional differences [81]. Contrary to the first premolar, maxillary second premolars mainly had single root (81.2-85.2%), two canals (52.7-61%) and type I canal configuration (29.7-70.8%) [55–57, 79, 84, 85]. However, two roots (10.4-15.8%) [56, 57, 81] and to some extent three roots (1.0%) were also present [56]. These results are justifiable as similar findings were obtained by another study [113] which was performed on different population of the world using CBCT. These results found that maxillary first premolar of Saudi population had higher evidence of two roots and canals (53.6%, 66.4%) than one root and canal (43.7%, 5%) along with presence of small percentages of three roots and canals (2.7%, 3.3%). However, single root and one canal was dominant in maxillary second premolar (90%, 60%) than two roots and canals (9.3%, 17.6%) and there was evidence of three root and more than two canals (0.7%, 0.7%). It was also observed that variations in the root and canal morphology of maxillary premolars were found among different countries which are linked to variations in geographic region, age, gender, and ethnicity [113]. Micro-CT study was done in an eastern province of Saudi Arabia [89], where detailed description of maxillary second premolar is provided including accessory canals and inter-canal communication as described in (Table 4).

Generally, mandibular premolars are single rooted, and their morphological variations are less. Mandibular first premolars had single root (80-99.5%), single canal (62.9-94.7%) and Vertucci’s type I configuration (62.9-95.1%) along with certain variations [32, 58, 60–62, 81, 84, 87]. Similarly, majority of second premolars were also found to be single rooted (80-100%), with one canal (71-98.6%) and type I canal configuration (91-97.9%) [32, 58–61, 81, 84]. There was no evidence of C-shaped root canals in mandibular first and second premolar, however C-shaped configuration was found in both the premolars in one study of Jazan city, this could be due regional differences in the selected sample size [83]. Ethnic diversity can contribute to variations in root canal morphology. Studies have shown that certain ethnic groups may have a higher prevalence of specific anatomical variations. This can be explained by a study of Martin et al. where prevalence of lingual canals in mandibular premolars was examined among population of different countries, there was presence of two canals (17%) and more than two canals (1%) in mandibular first premolar of Saudi population. Similarly two canals (3.3%) and more than two canals (1.3%) were also found in mandibular second premolar [114]. Therefore, it is essential to consider the ethnic background of the population being studied when interpreting results and making clinical decisions [115].

Maxillary first molars are often endodontically treated teeth as a result of variations in their canals, there can be evidence of separated roots or fused roots and commonly missed second mesiobuuccal canal challenges the treatment procedure [7]. Three roots variant was the most common (90-100%) compared to four roots (6-9.8%) [63, 67, 68, 82, 88]. Similarly, the three canals were more frequent (40-86.8%). However, there were evidence of two canals (3.7%), 4 canals (21-55.6%) [63, 65, 67, 82]. Presence of MB root, MB and MB2 canal (80-86.8%) was also noticed among the included studies of different regions [65, 82, 88].

Likewise, maxillary second molars were single rooted (0.3%), double rooted (2.7-6.6%), three rooted (92-99.3%) and four rooted (0.75-1.1%) and they also revealed the presence of MB root [64, 67–69]. The prevalence of MB2 canals in maxillary first molars exhibited significant variation in the available literature. Prior research mostly concentrated on the morphology of roots and intricate canal shapes, as well as the influential elements of respondents’ sex, age, and ethnicity. However, limited research has been conducted on the correlation between the existence of an MB2 canal and the arrangement of their apical portals of exit [116].

More often mandibular molars have two roots (89.6-97.1%), three canals (64.5-78.9%) but there can be evidence of three (9.8%) or four roots (2.9%) along with two or four canals and type I configuration being predominant in distal root whereas type IV in mesial roots. They also exhibit fusion of roots and discrete canal configuration. Further C- shaped canals and inter canal communication was also noticed [70, 71, 73, 74, 82]. In addition to this, radix molaris (4.5%) in which, radix entomolaris was (4.2%) and radix paramolaris was (0.3%) in mandibular first molars were found in the Riyadh and Al-Kharj region [75]. One study revealed the lower prevalence of radix entomolaris, and a second canal in the distal root of mandibular first molars among Saudi Arbian population compared to other populations in the East Asia geographic region and Asian ethnic groups, which had higher prevalence of radix entomolaris and extra canal in the distal root of mandibular first molars [117].

### Strength

This systematic review involved all CBCT and microCT studies that examined the root and canal anatomy in Saudi Arabian population.

## Limitations

This systematic review included all studies without restrictions related to the voxel size of the CBCT images, which could affect the comparisons among different studies [118].

## Future directions

- Future research in this field should aim to address demographic factors and their potential impact on root canal anatomy in Saudi Arabian population.
- In future research, the integration of micro-CT and CBCT data could provide a more comprehensive understanding of root canal anatomy. Comparative studies that evaluate the accuracy and diagnostic capabilities of these imaging modalities in clinical practice would be valuable.
- Future research in the field of endodontics should consider the influence of ethnicity, age, and gender on root canal anatomy in different Saudi Arabian sub-populations. Furthermore, the integration of advanced imaging modalities.
- Clinicians should be aware of the potential anatomical variations they may encounter and consider the use of advanced imaging techniques when necessary to improve the quality of endodontic treatment.
- Sample size calculation has to be considered in future studies related to root and canal anatomy in Saudi Arabian population.
- The application of Ahmed et al. classification system can also be used in future studies. A recent systematic review showed that the literature supports the advantages of the Ahmed et al. system, compared to other systems [119].
- The use of PROUD reporting guidelines [46] for root and canal anatomy studies can also be helpful to standardize the reporting of study parameters and specifications.

## Conclusion

In conclusion, this systematic review highlights the significant variations in root canal morphology within the Saudi Arabian population. These variations can be attributed to genetic, environmental, and regional factors, as well as differences in imaging techniques and demographic characteristics. CBCT and micro-CT was used in the present review to give knowledge about the root canal morphology of permanent dentition among different regions of Saudi population. It can be concluded from the study that widespread variations exist concerning the root canal anatomy, hence careful examination by suitable method is necessary. Mandibular incisors, mandibular premolars followed by maxillary molars were more commonly examined teeth among the included studies. Both the methods used were efficient in providing data of normal and altered root and canal anatomy of teeth with significant information.

## Data Availability

All data from the published papers are included in the current study.
